# Characterization of HERV-K (HML-2) Rec proteins encoded in the human genome and their post-transcriptional function

**DOI:** 10.1128/jvi.01515-25

**Published:** 2025-11-18

**Authors:** Katarzyna Zurowska, Godfrey Dzhivhuho, David Grabski, David Rekosh, Marie-Louise Hammarskjold

**Affiliations:** 1 Myles H. Thaler Center for AIDS and Human Retrovirus Research, School of Medicine, University of Virginia 12349, Charlottesville, Virginia, USA; 2 Department of Microbiology, Immunology, and Cancer Biology, School of Medicine, University of Virginia 174484, Charlottesville, Virginia, USA; 3 Department of Surgery, School of Medicine, University of Virginia 346215, Charlottesville, Virginia, USA; University Hospital Tübingen, Tübingen, Germany

**Keywords:** endogenous retroviruses, protein diversity, RNA export, HERV-K

## Abstract

Human endogenous retrovirus K (HERV-K) proviruses of the HML-2 subgroup are the most recently integrated retroviral elements within the human genome. The HERV-K Rec protein, a functional homolog of HIV Rev, is essential for the expression of viral mRNAs with retained introns. However, their diversity and functional capacities have remained largely unexplored. We identified Rec coding sequences in the human genome and selected intact Rec proteins from 28 proviral loci for functional characterization. Using a dual-color fluorescent reporter containing the HERV-K Rec-response element and a complementary enzyme-linked immunosorbent assay, we found that Rec proteins from nine proviral genomic loci promoted function at the post-transcriptional level. All but one of these proviruses are insertionally polymorphic in the human genome. In addition, several of the non-functional Rec proteins were trans-dominant negative. Detailed mutational analysis of the most potent inhibitory variant, encoded by the HERV-K provirus 12q14.1, displayed only two amino acid changes (N2H and E34del) relative to the prototypical functional Rec protein encoded by the reconstructed consensus HERV-K. Insertion of a glutamic acid at position 34 in this mutant fully restored the functional activity. Our results unveil an unexpected complexity in HERV-K (HML-2) post-transcriptional regulation, with a few Rec variants showing function, while others are trans-dominant negative. These findings significantly expand our understanding of the various Rec proteins that can be expressed in human cells and how they function at the post-transcriptional level.

**IMPORTANCE:**

In this study, we compared Rec sequences from 58 type 2 human endogenous retrovirus K proviruses and showed that only 9 encode Rec proteins that are functional in post-transcriptional regulation of RNAs with retained introns. We also showed that several Rec proteins have trans-dominant negative activity when co-expressed with a functional Rec protein. While previous studies have demonstrated the expression of Rec mRNAs and proteins in human cells, this is the first study to define which loci have the potential to encode either functional or trans-dominant negative Rec proteins. Additionally, our reporter system will also enable future investigations to easily determine whether functional and/or trans-dominant negative Rec proteins are expressed in any cell. These findings are also important for future studies that aim to link Rec post-transcriptional function to physiological or pathological effects.

## INTRODUCTION

Human endogenous retroviruses (HERVs), which constitute approximately 8% of the human genome, are a class of transposable elements that are remnants of ancient exogenous retroviruses. These viruses integrated into the primate germline between 100 and 40 million years ago (1, 2). Although HERVs have accumulated many mutations and deletions within coding sequences over time (3), many remain transcriptionally active, contributing to cellular function either through the production of viral proteins or by serving as regulatory elements for host gene expression (3–6).

Among HERVs, the HERV-K (HML-2) subgroup is the most recent and biologically active. While most HERV-K proviruses in the human genome are shared with other great apes and date back 5–30 million years, a subset is human-specific and polymorphic. Notably, the insertion of HERV-K115 has been estimated to have occurred as recently as ~350,000 years ago (5), indicating that this retroviral family remained active well into recent human evolutionary history.

HERV taxonomy is based on sequence similarity to other animal retroviruses (7–9). The HERV-K clade belongs to class II of beta retrovirus-like endogenous retroviruses, with the “K” designation referring to the lysine tRNA primer used to prime reverse transcription in these viruses. The HERV-K (HML-2) subgroup that comprises more than 100 proviruses is the most recently integrated and biologically active. In this report, HERV-K (HML-2) virus will hereafter be referred to simply as HERV-K.

Many HERV-K copies retain intact open reading frames (ORFs) with the potential to produce viral RNAs and, in many cases, viral proteins. Complete HERV-K genomes, like all intact retroviruses, encode the essential gag, pro, pol, and env genes, flanked by long terminal repeats (LTRs) (7). Additionally, like the more complex viruses, such as HTLV-1 and HIV (10), they also encode regulatory proteins (11). HERV-K proviruses are classified as type 1 or type 2. Type 2 proviruses encode the Rec protein from a doubly spliced transcript; type 1 proviruses have mutations and a 292-bp deletion in the pol-env region, which deletes the first coding exon of *rec* and changes the doubly spliced transcript in some of the type 1 viruses, to encode a protein named Np9 (7, 11–14).

Rec interacts directly with the Rec-Response Element (RcRE), present in the 3′ end of all HERV-K mRNAs (11, 15, 16). Through this interaction, Rec facilitates the nucleocytoplasmic export and expression of HERV-K mRNAs that contain retained introns, using the Crm1-RanGTP pathway (11, 17). Rec contains an arginine-rich nuclear location signal (NLS), as well as a nuclear export signal (NES) (17, 18), and functions similarly to the HIV Rev, HTLV Rex, and mouse mammary tumor virus (MMTV) Rem proteins (19–24). In addition to the proviruses, more than 900 HERV-K solitary LTRs are scattered throughout the human genome (5). Many of these contain RcRE sequences that can potentially interact with Rec proteins. Recent studies have identified cellular genes that contain sequences with high homology to HERV-K RcREs, suggesting that Rec may modulate cellular gene expression through interactions with these elements (5, 25–28).

Some evidence suggests that HERV-K Rec may play significant roles in cancer development, potentially through interactions with cellular proteins. For example, Rec has been shown to interact with the promyelocytic leukemia zinc-finger protein (PLZF), activating c-myc proto-oncogene expression and promoting cell proliferation (13, 29). Rec has also been shown to interact with the human small glutamine-rich tetratricopeptide repeat-containing protein (hSGT). This leads to increased androgen receptor activity, which may promote oncogenesis (30).

The first coding exon of Rec is the same as the first 87 amino acids of the 95 amino acid Env signal peptide (SP) (31). The second coding exon also overlaps *env* sequences but is translated in a different open reading frame. In the case of the closely related MMTV, it has been shown that the Env SP, which corresponds to the first coding exon of the nuclear export protein Rem, is sufficient to function in the nuclear export of viral mRNAs (32) since it contains RNA binding, as well as NLS and NES domains. The HERV-K Env SP also contains the NLS and NES sequences, which suggests that it could also be sufficient for HERV-K mRNA export. This relationship and the fact that both coding exons of Rec overlap the *env* coding region become particularly relevant when interpreting studies showing oncogenic properties of HERV-K Env in various cancers (33, 34), as Env expression is also likely to depend on Rec expression. Even though the *env* mRNA is spliced, it still retains an intron, similar to Env mRNAs in other complex retroviruses, which have been shown to require a *trans*-acting protein to facilitate nucleo-cytoplasmic export and expression (10, 19–24).

Despite the widespread distribution of HERV-K sequences in the human genome and the potential importance of Rec in regulating viral and cellular gene expression, an analysis of which proviral loci produce Rec proteins that can function at the post-transcriptional level has not been performed. In this study, we performed a comprehensive functional analysis of the HERV-K Rec proteins that can be expressed from type 2 proviral loci. Our findings reveal that while some Rec variants promote the expression of RcRE-RNA with retained introns, others act as trans-dominant negative regulators, suggesting that post-transcriptional Rec regulation of HERV-K expression is complex.

## RESULTS

### Characterization and selection of HERV-K HML-2 Rec proteins for functional analysis

**Fig 1:**
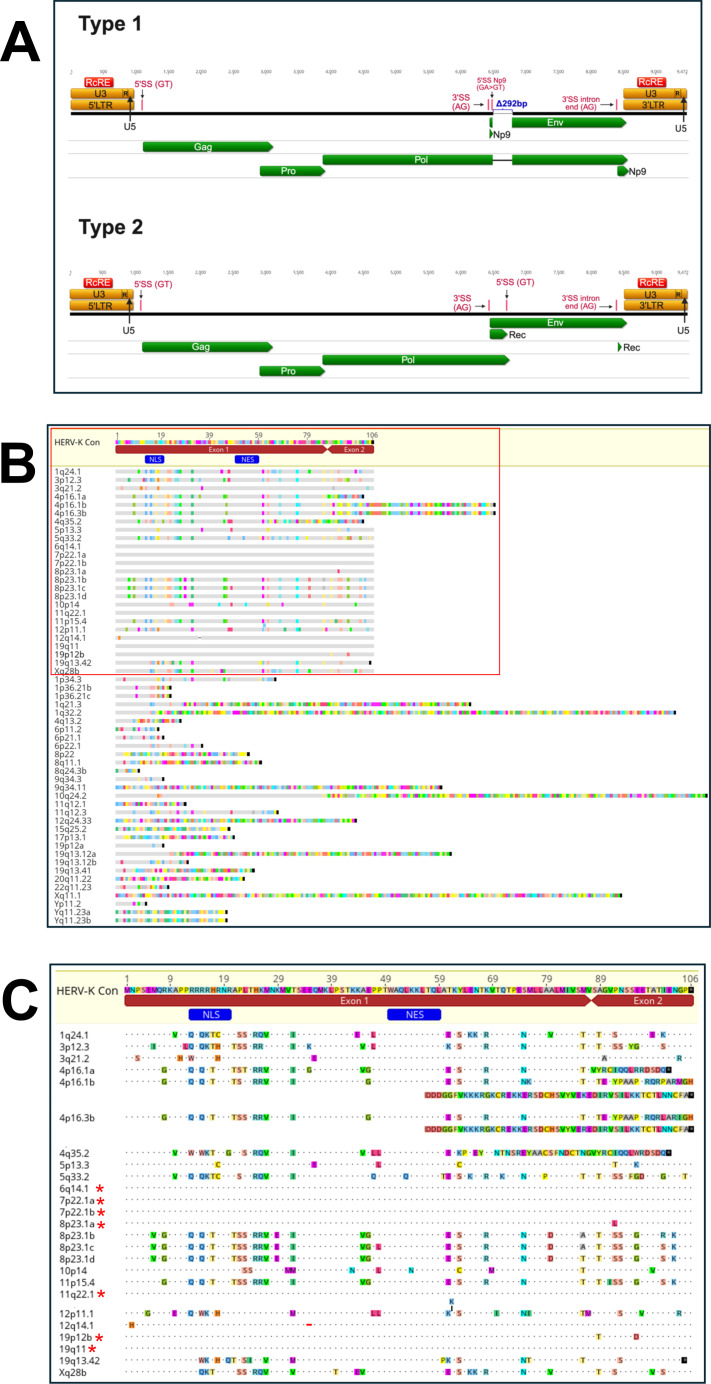
Bioinformatic characterization and selection of HERV-K HML-2 Rec proteins for functional analysis (from the proviruses listed in reference 5). (**A**) Diagram of the primary features of the HERV-K Con provirus, including LTRs, ORFs, and specific splice sites. This schema was used for the annotation of type 1 (modified version with a 292-bp deletion in the pol-env junction: top panel) and type 2 (the original HERV-K Con: bottom panel) proviral reference genomes. Relative to type 2, there is an A to T point mutation in many type 1 proviruses that introduces a stop codon in the env reading frame. This also creates a new 5′ splice site, which allows for the generation of a doubly spliced mRNA that encodes the Np9 protein. Eight bases downstream from the splice site, the 292-bp deletion removes most of the first coding exon of rec. A downstream methionine in type 1 viruses may allow for translation of an N-terminally truncated Env protein. This protein would not be expected to be glycosylated, as it lacks a signal peptide. (**B**) Alignment of translated Rec coding sequences (extracted and joined using the known splice sites in type 2 proviruses) to the HERV-K Con Rec protein. This yielded 55 distinct Rec protein sequences. Differences relative to the HERV-K Con Rec protein sequence are highlighted in colored blocks. Gray line represents identical amino acid residues, while the dash in 12q14.1 indicates an amino acid deletion. Stop codons are represented as black boxes. A red box highlights the subset of Rec sequences that were experimentally tested. Rec sequences outside the red box were excluded from testing due to extensive mutations and/or premature stop codons. Annotations (blue) show the NLS and NES. Annotations (red) show coding exons 1 and 2. (**C**) Comparative sequence analysis of the 25 full-length Rec protein sequences highlighted in the red box in panel B. Differences from the HERV-K Con Rec sequence are colored; dots represent identity with the reference sequence; the red line indicates a deletion in 12q14.1; black boxes with asterisks (*) mark stop codons. For 4p16.1b and 4p16.3b loci, long Rec sequences are displayed across two lines for readability. For 12p11.1, a lysine (K) insertion at position 62 is indicated by a vertical black line at that position. Annotations (blue) show the NLS and NES. Annotations (red) show coding exons 1 and 2. Red asterisks (*) indicate loci that encode functional Rec proteins. A summary of all the tested Rec proteins is included in Table S5.

The identification and characterization of functional HERV-K HML-2 Rec proteins present significant challenges due to the repetitive nature and abundance of HERV sequences in the human genome. We approached this problem by first annotating key features of the reconstituted HERV-K provirus, HERV-K Con, including long terminal repeats, open reading frames, and known splice sites (Fig. 1A) (35, 36). To account for both type 1 and type 2 proviruses, we generated two reference genomes: the original HERV-K Con (type 2) and a modified version with a 292-bp deletion in the pol-env region (type 1). We then aligned 91 HERV-K genomes described in Subramanian et al. (5) to these annotated references, transferring annotations to each sequence. This process enabled us to differentiate between type 2 proviruses (55 copies) containing the *rec* gene and type 1 proviruses with the 292-bp deletion. Table S4 shows all of the type 2 HERV-K loci analyzed in this paper, along with their chromosomal positions in the hg38 genome and their corresponding commonly used names (where available), such as HERV-K113 and HERV-K108. The loci from which Rec sequences were tested are highlighted in red.

Our overall strategy was informed by prior analyses of Rec, which cataloged several Rec open reading frames and their distribution across the HERV-K (HML-2) family (37, 38). However, these studies did not include an analysis of the functional activity of the predicted Rec proteins.

To create the potential Rec proteins that could be expressed from each of the 55 loci and assayed for functional activity, we first determined if the 5′ and 3′ splice sites connecting the two *rec* exons were intact. If they were, we spliced the two exons together. If either splice site was not intact, we kept the first coding exon contiguous with the *env* sequence. These sequences are now included in FASTA format in File S1. Each sequence was then translated to determine the presence of stop codons and indels leading to frameshifts. The translated protein sequences are shown in Fig. 1B and C, and the sequences in FASTA format are listed in File S2. In performing our analysis, we found that the 3′ splice site upstream of the second coding exon of Rec in provirus 10q24.2 is mutated from AG to GG, and thus, the predicted protein sequence does not include a proper second exon. For the loci that could potentially produce functional Rec proteins, we also examined the major 5′ splice site and the 3′ splice site just upstream of the first coding exon of Rec. In all cases, these splice sites remain intact. Additionally, except for 19q11, all proviruses have mostly intact 5′ LTRs. However, expression of this provirus has been previously reported (39), suggesting that an upstream cellular promoter may be used for transcription.

This alignment revealed striking protein sequence diversity among the Rec proteins encoded by these HERV-K loci (Fig. 1B). Over half of the ORFs were truncated due to large deletions and/or premature stop codons. We identified and selected 25 proviruses that could express full-length Rec proteins, excluding 29 incomplete or heavily truncated protein sequences, as well as the 10q24.2 provirus that lacks the 3′ splice site for the second coding exon, from functional analysis. Among the selected 25 full-length proteins, we observed significant sequence diversity compared to the reference HERV-K Con Rec (Fig. 1C). However, five Rec proteins (encoded by proviruses 6q14.1, 7p22.1a, 7p22.1b, 11q22.1, and 19q11) were identical to the reference HERV-K Con Rec at the protein level, despite one to three nucleotide differences in their coding sequences (36). Thus, we initially selected 21 distinct Rec protein variants for the analysis of post-transcriptional function: 1 identical to the reference HERV-K Con Rec and 20 with amino acid variations.

### Evaluation of HERV-K HML-2 Rec protein post-transcriptional function using an RcRE-reporter assay

**Fig 2:**
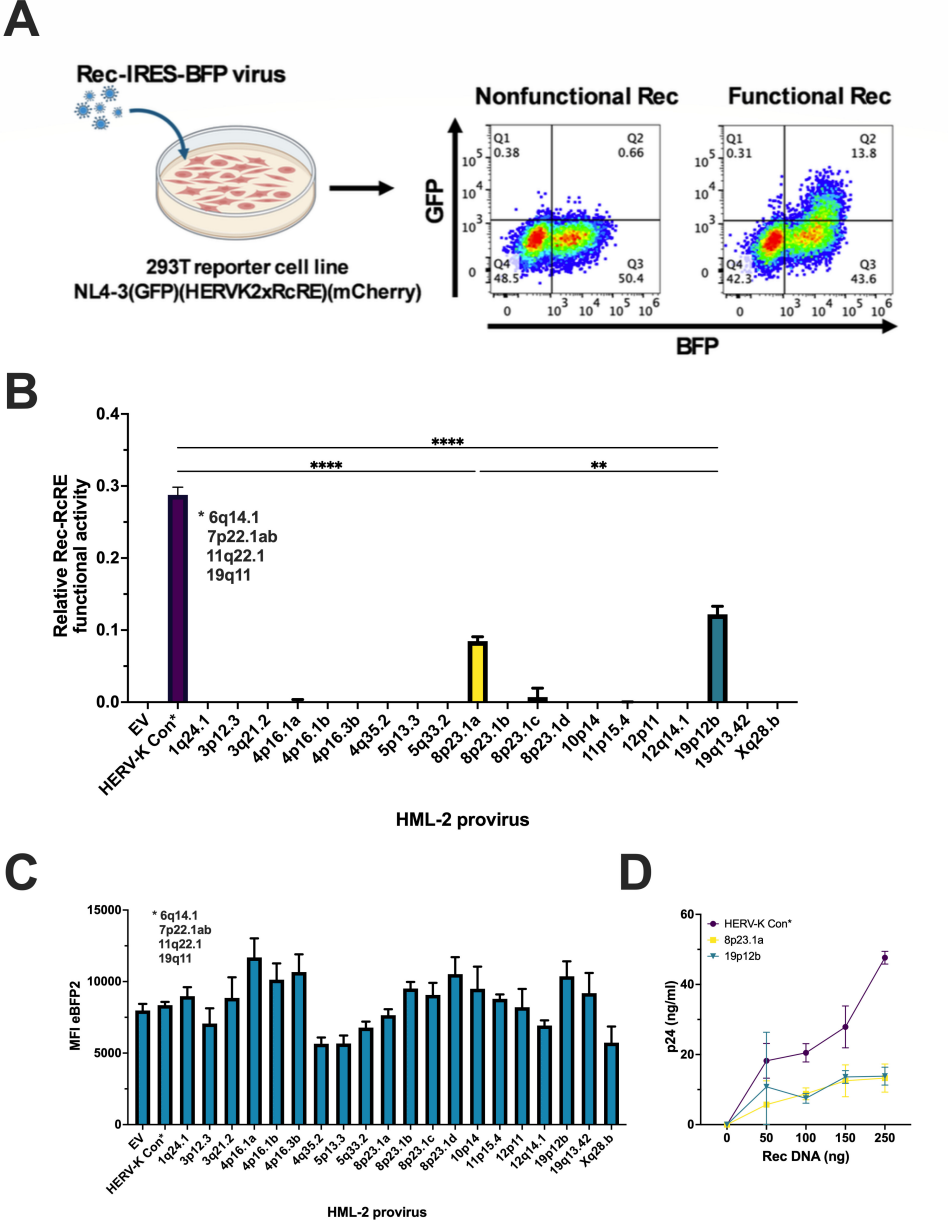
Functional analysis of HERV-K Rec proteins. (**A**) Schematic of the dual-color Rec-reporter assay using retroviral vectors expressing Rec and eBFP2. The cells contain a reporter vector that constitutively produces mCherry from a fully spliced transcript. Upon transduction with the Rec-expressing vectors, the mCherry-positive cells also express eBFP2. Functional Rec proteins interact with the RcRE, promoting nuclear export of the unspliced mRNA encoding GFP (right flow chart). Non-functional Rec proteins result in only eBFP2 expression (left flow chart). (**B**) Quantification of Rec functional activity using the dual-color reporter assay by flow cytometry analysis performed 72 hours post-transduction. 293T/17 RcRE-reporter cells were transduced with vectors expressing the various Rec proteins and eBFP2. Rec activity was assessed by gating on mCherry-positive cells, selecting eBFP2-positive cells (indicating Rec vector expression), and measuring GFP and eBFP2 expression. Rec/RcRE activity was calculated as the ratio of GFP mean fluorescence intensity to eBFP2 mean fluorescence intensity. The HERV-K Con Rec sequence represents five identical Rec proteins (6q14.1, 7p22.1a, 7p22.1b, 11q22.1, and 19q11) (marked with an asterisk). The data are shown as the mean value ± SD from three independent experiments. Significant differences were assessed by ordinary one-way ANOVA with Tukey’s *post hoc* test (**P < 0.05 and ****P < 0.0001). (**C**) eBFP2 expression levels from the transductions performed in panel **B**. The mean fluorescence intensity (MFI) of eBFP2 expressed from each of the individual transductions was determined using flow cytometry. Data are shown as mean ± SD from three independent experiments. (**D**) Validation of Rec activity using a p24 release assay. 293T/17 cells were co-transfected with equal amounts of a Gag-Pol-RcRE reporter vector (1,500 ng) and increasing amounts of functional Rec vectors (50, 100, 150, and 250 ng). An empty vector was used to normalize the total DNA input to 2,000 ng. Supernatants were collected at 72 hours post-transfection, and p24 was then measured by enzyme-linked immunosorbent assay. The HERV-K Con Rec sequence represents five identical Rec proteins (6q14.1, 7p22.1a, 7p22.1b, 11q22.1, and 19q11) (marked with an asterisk). The data shown are the mean values ± SD from three independent experiments.

To evaluate the functional capacity of the selected HERV-K HML-2 Rec proteins, we developed an assay using a dual-color fluorescent reporter vector. This vector is similar to our established HIV-1 Rev-RRE dual-color reporter but contains a tandem RcRE in place of the HIV RRE (40, 41). This modified reporter produces mCherry constitutively from a spliced mRNA, while GFP expression from an unspliced RNA depends on the presence of both the *cis*-acting RcRE and a functional *trans*-acting Rec protein. For a diagram of this vector, see Fig. S1. To facilitate our experiments, we generated a 293T/17-based cell line stably expressing the reporter mRNA (Fig. 2A).

To test the activity of the various Rec proteins, we cloned the ORFs for each of the 21 proteins into a murine stem cell virus-derived retroviral vector (pMSCV), positioning them upstream of an IRES-eBFP2 cassette. This bicistronic design ensures simultaneous expression of both Rec and eBFP2 from a single transcript, facilitating detection and quantification of expression levels.

If a functional Rec protein is expressed from this vector in the transduced reporter cells, its interaction with the results in the export of the unspliced mRNA and GFP expression. After flow cytometry analysis, Rec functional activity can be quantified as the ratio of GFP mean fluorescence intensity (MFI) to eBFP2 MFI. By using this ratio, the results are normalized to account for potential differences in transduction efficiency and RNA expression, since Rec and eBFP2 are translated from the same mRNA.

Transduction experiments with the vectors expressing the 21 different Rec proteins showed that, in addition to HERV-K Con Rec, encoded by the five proviruses at 6q14.1, 7p22.1a, 7p22.1b, 11q22.1, and 19q11, only two other Rec proteins (at loci 8p23.1a and 19p12b) displayed functional activity (Fig. 2B). All but one of these Rec proteins (19q11) originate from proviral loci that are polymorphic in the human genome. 19q11 lacks the 5′ LTR, but viral RNA may be transcribed from an upstream promoter, since expression from this provirus has been reported in the literature (39). The remaining Rec variants showed no significant activity, as indicated by the absence of GFP expression despite eBFP2 expression (Fig. 2C). We also employed a complementary approach, using an HIV GagPol-RcRE reporter vector to further validate and quantify the activity of the functional Rec protein variants. This reporter measures p24 release as an indicator of viral protein expression mediated by a functional Rec/RcRE interaction. To perform this assay, the Gag-Pol-RcRE reporter was co-transfected into cells with increasing amounts of the plasmids expressing the Rec proteins (Fig. 2D). This confirmed the functionality of the three protein variants.

**Fig 3:**
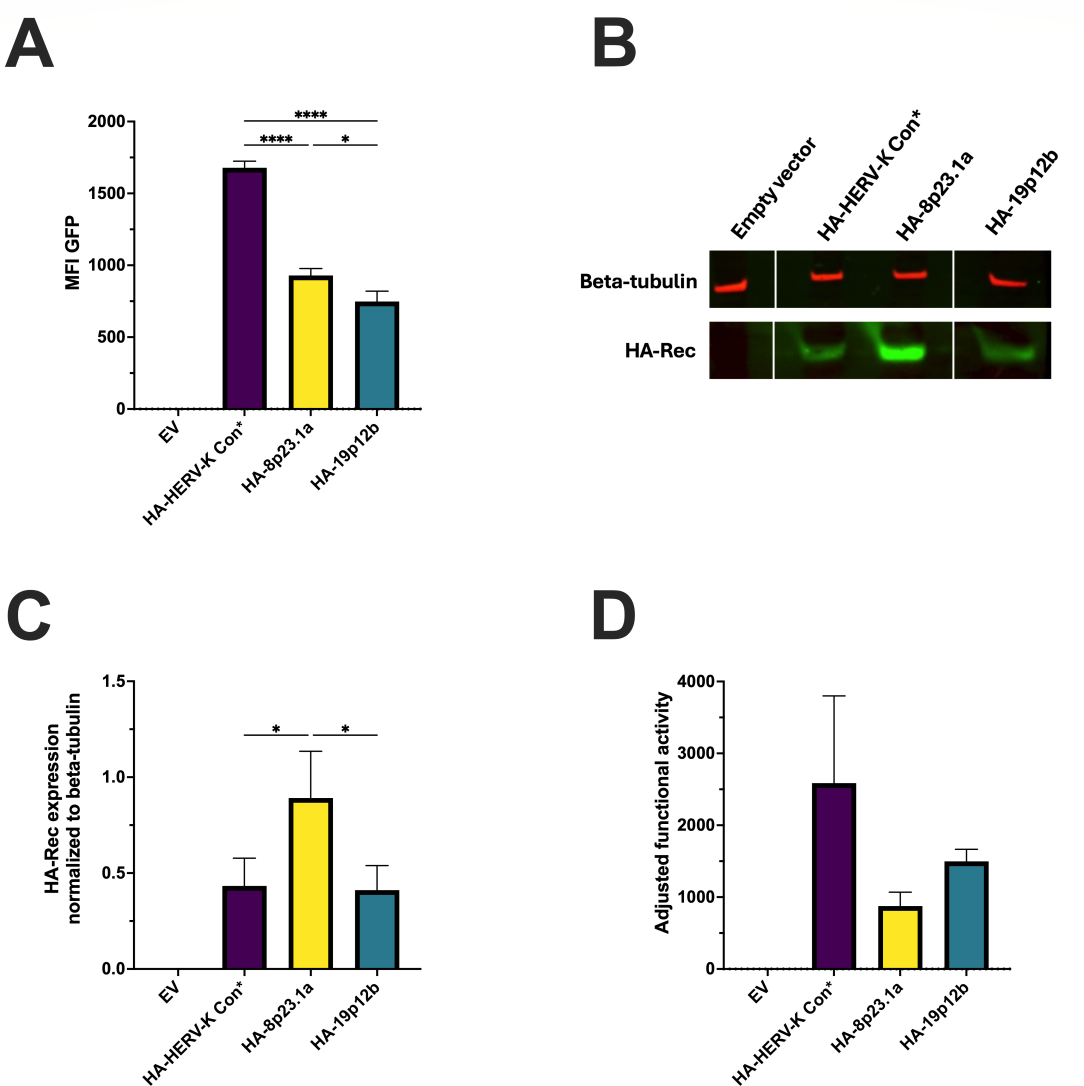
Functional analysis and quantitation of HA-tagged Rec proteins (**A**) Functional activity of HA-tagged Rec variants. 293T/17 RcRE-reporter cells were transfected using Lipofectamine 3000 with vectors expressing the various Rec proteins. GFP MFI was measured 72 hours post-transfection as an indication of Rec functional activity. HERV-K Con Rec (marked with an asterisk) represents five identical Rec protein sequences (6q14.1, 7p22.1a, 7p22.1b, 11q22.1, and 19q11). Statistical significance assessed by ordinary one-way ANOVA with Tukey’s *post hoc* test (*P < 0.05 and ****P < 0.0001). (**B**) Western blot analysis of HA-tagged Rec protein expression. Upper panel: β-tubulin loading control; lower panel: HA-tagged Rec detection. Representative Western blot from two independent experiments using two different plasmid preparations. (**C**) Quantification of HA-tagged Rec protein expression levels normalized to β-tubulin loading control. Data represent the mean value ± SD from two independent experiments with triplicate samples using two different plasmid preparations. Statistical significance was assessed by ordinary one-way ANOVA with Tukey’s *post hoc* test (*P < 0.05). (**D**) Functional activity of HA-Rec variants normalized to protein expression. Rec/RcRE functional activity (from panel A) was normalized to HA-tagged protein expression levels (**C**) by calculating the ratio of GFP MFI to normalized HA-Rec protein levels. Data represent the mean value ± SD from two independent experiments with triplicate samples using two different plasmid preparations.

We next decided to directly investigate how functional activity was related to steady-state levels of Rec expression. Due to the lack of commercially available Rec antibodies, we generated constructs with HA tags added to the N-termini of these Rec proteins, enabling visualization of their expression by Western blot using an anti-HA antibody. As shown in Fig. 3A, all three of the tagged constructs displayed Rec activity. Western blot analysis showed some differences in protein expression levels among the HA-tagged Rec variants (Fig. 3B and C). We thus normalized the Rec activity based on the protein expression levels (Fig. 3D). These assays demonstrated that HERV-K Con displayed the highest activity, as was also demonstrated in the transduction experiments.

### Some HERV-K HML-2 Rec proteins are trans-dominant negative

**Fig 4:**
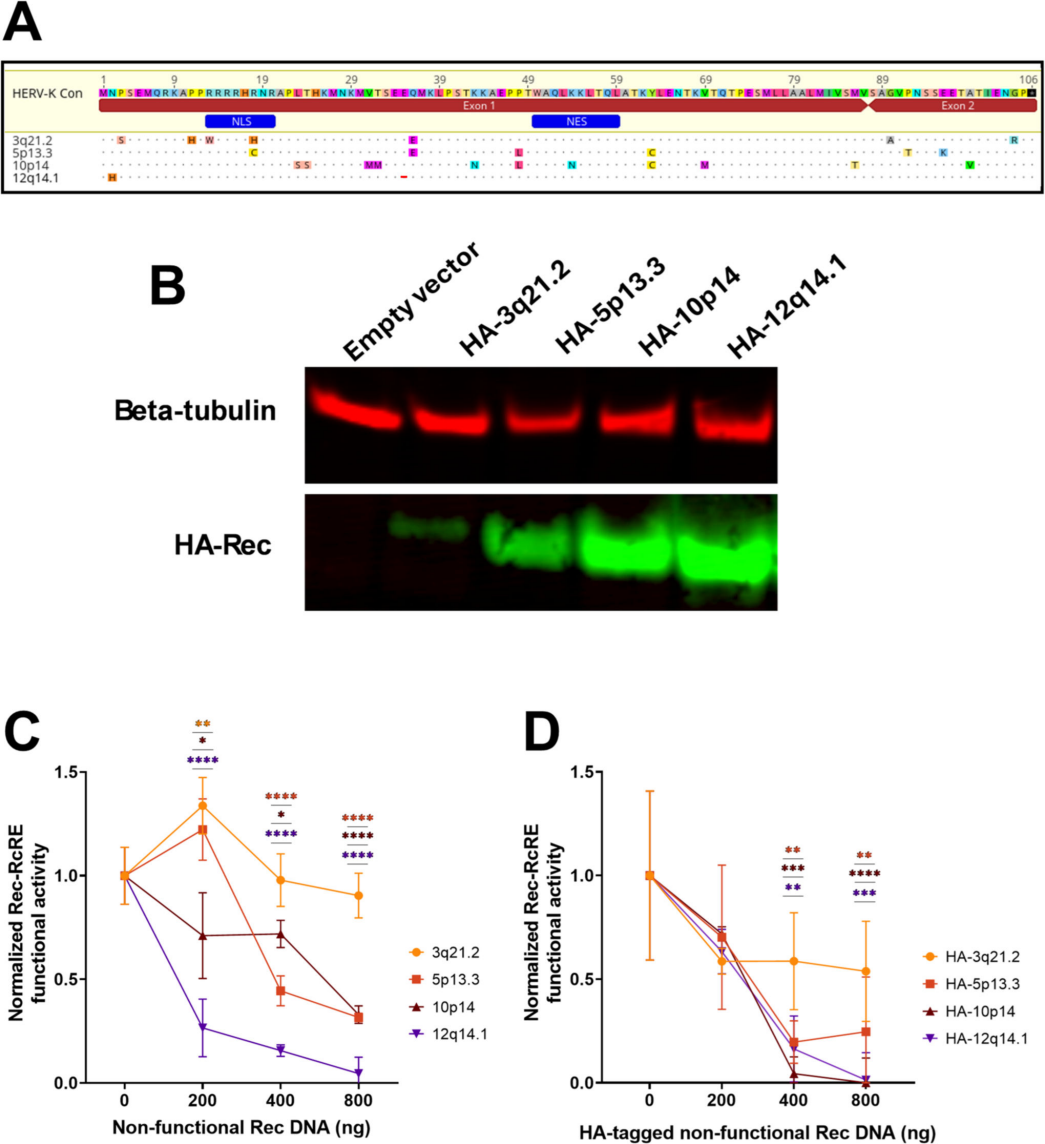
Analysis of selected non-functional HERV-K Rec proteins and their trans-dominant negative effects. (**A**) Multiple sequence alignment of selected non-functional Rec proteins (3q21.2, 5p13.3, 10p14, and 12q14.1) to the reference HERV-K Con Rec. Selected variants contain fewer than 15 amino acid changes compared to the consensus sequence. Amino acid differences are highlighted in color and shown in uppercase letters. Dots indicate residues identical to HERV-K Con Rec; the red dash in 12q14.1 represents an amino acid deletion. Position numbers correspond to the HERV-K Con Rec sequence. Annotations (blue) show the NLS and NES. Annotations (red) show coding exons 1 and 2. (**B**) Western blot analysis of non-functional Rec protein expression. Upper panel: β-tubulin loading control; lower panel: HA-tagged Rec protein detection. A Western blot representative of two independent experiments using two different plasmid preparations is shown. (**C**) Trans-dominant negative activity analysis using untagged Rec variants in 293T/17 reporter cells. Cells were co-transfected with a constant amount of HERV-K Con Rec (100 ng) and increasing amounts (0–800 ng) of non-functional Rec-expressing plasmids, maintaining total DNA at 2,000 ng with empty vector. Cells were also transfected with an empty vector to determine background GFP expression. Control samples received 100 ng HERV-K Con Rec plus 1,900 ng empty vector. GFP expression was measured by flow cytometry 72 hours post-transfection. The GFP background from the empty vector samples was subtracted, and the data were normalized, with the control sample set to 1.0. Rec/RcRE functional activity was plotted as mean fluorescence intensity of GFP. Data represent the mean value ± SD from three independent experiments. Statistical significance was assessed by ordinary two-way ANOVA with Dunnett’s multiple comparisons *post hoc* test (*P < 0.05, **P < 0.01, and ****P < 0.0001). (**D**) Trans-dominant negative activity analysis using HA-tagged Rec variants in 293T/17 RcRE reporter cells. Cells were co-transfected with HA-HERV-K Con Rec (100 ng) and increasing amounts (0–800 ng) of HA-tagged non-functional Rec variants. Data were collected, analyzed, and normalized as described in panel C. Data represent the mean value ± SD from three independent experiments. Statistical significance was assessed by ordinary two-way ANOVA with Dunnett’s multiple comparisons *post hoc* test (**P < 0.01, ***P < 0.001, and ****P < 0.0001).

We hypothesized that some of the non-functional Rec proteins might display a trans-dominant negative phenotype. To test this, we selected the four non-functional Rec proteins (encoded at 3q21.2, 5p13.3, 10p14, and 12q14.1) that were the least changed (<15 aa changes) compared to HERV-K Con Rec (Fig. 4A). We cloned cDNA copies of each coding sequence, with or without N-terminal HA-tags, into expression plasmids. The HA-tagged plasmids were then transfected into the RcRE-reporter cell line. Western blot analysis revealed that all four selected non-functional Rec proteins were expressed in these cells, but the Rec protein encoded by provirus 3q21.2 showed only very low levels of expression (Fig. 4B).

To analyze if these Rec proteins were trans-dominant negative, we next co-transfected increasing amounts (0–800 ng) of either the tagged or the non-tagged plasmids into RcRE-reporter cells, with a constant amount (100 ng) of the plasmid expressing the active non-tagged HERV-K Con Rec. An appropriate amount of empty vector was added to maintain a total of 2 µg of transfected DNA. We then measured Rec functional activity using flow cytometry.

In the experiment with both the non-tagged and HA-tagged Rec proteins, three of the four non-functional Rec proteins (5p13.3, 10p14, and 12q14.1) exhibited some degree of trans-dominant negative effect (Fig. 4C and D). However, Rec 3q21.2 showed no significant inhibitory effect. Interestingly, the 12q14.1 Rec protein that displayed a very potent trans-dominant negative effect had only two changes compared to HERV-K Con Rec: an N2H mutation and a deletion at position 34 (E34del) (Fig. 4A).

### Mutational analysis of the trans-dominant negative Rec 12q14.1

**Fig 5:**
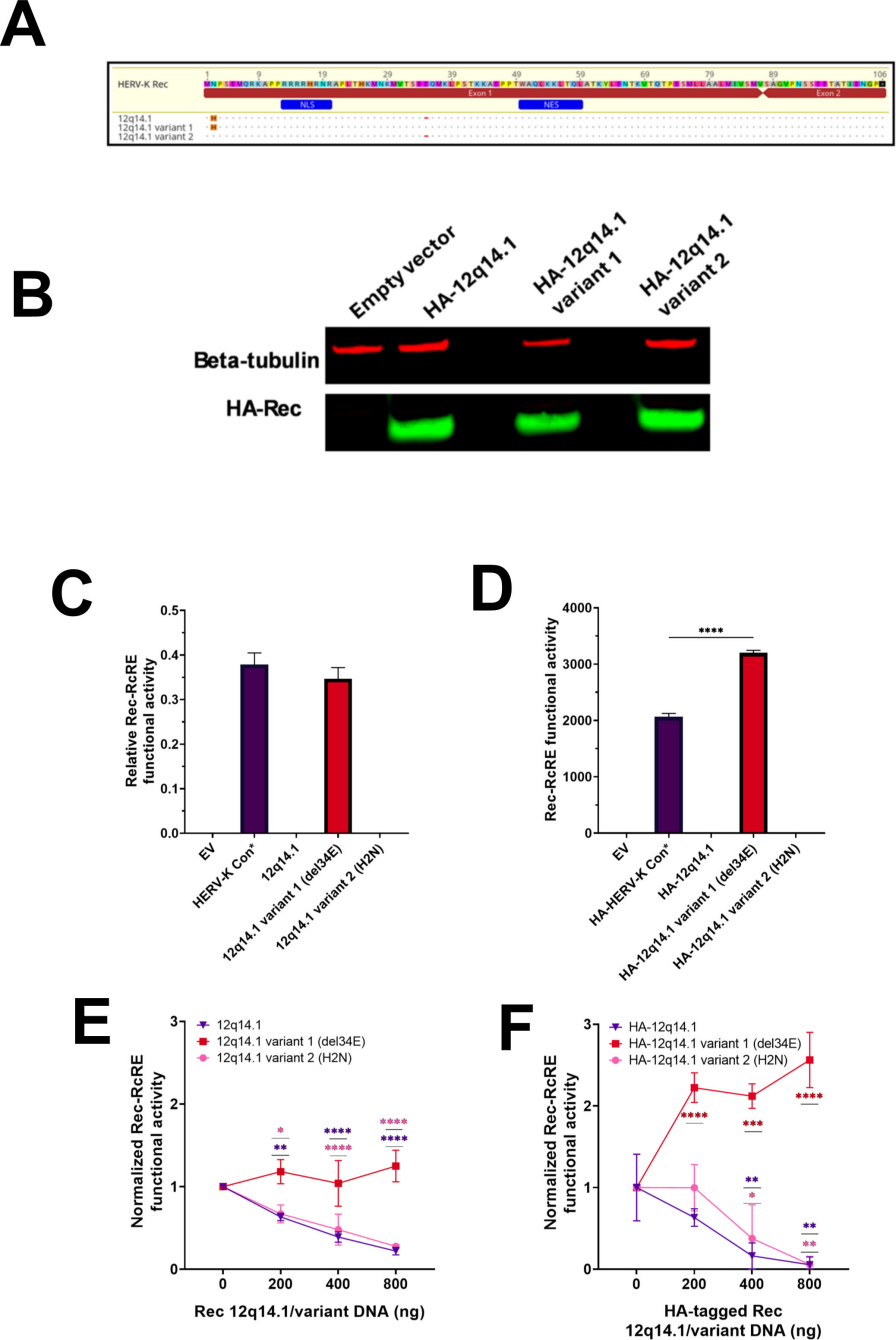
Mutational analysis of the trans-dominant negative Rec 12q14.1. (**A**) Sequence alignments of Rec 12q14.1 variants relative to the HERV-K Con Rec sequence. The alignments show the HERV-K Con Rec sequence, the original Rec 12q14.1, and the two generated variants. Amino acid differences are highlighted in color and shown in uppercase letters. Dots represent identical residues to the consensus sequence. The red dash indicates an amino acid deletion. Position numbers correspond to the HERV-K Con Rec consensus sequence. Variant 1 (del34E) and Variant 2 (H2N) are named relative to the parental 12q14.1. Functional domains are annotated in blue (NLS and NES). Annotations (blue) show the NLS and NES. Annotations (red) show coding exons 1 and 2. (**B**) Western blot analysis of Rec variant protein expression. Upper panel: β-tubulin loading control; lower panel: HA-tagged Rec protein detection. A Western blot representative of two independent experiments using two different plasmid preparations is shown. (**C**) Functional analysis of Rec variants in 293T/17 RcRE-reporter cells. Cells were transduced with retroviral vectors expressing each variant or HERV-K Con Rec (positive control). Fluorescent protein expression was then measured by flow cytometry 72 hours post-transduction. Rec/RcRE functional activity is plotted as the ratio of GFP MFI to eBFP2 MFI. Data represent the mean value ± SD from three independent experiments. Statistical significance was assessed using an unpaired *t*-test with Welch’s correction. (**D**) Functional analysis of HA-tagged Rec variants in 293T/17 2xRcRE reporter cells. Cells were transfected with retroviral vectors expressing each HA-tagged variant or HERV-K Con Rec (positive control). GFP expression was measured by flow cytometry 72 hours post-transduction. Rec functional activity was plotted as MFI of GFP. The data represent the mean value ± SD from three independent experiments. Statistical significance was assessed using an unpaired *t*-test with Welch’s correction (****P < 0.001). (**E**) Trans-dominant negative activity analysis using untagged Rec variants. 293T/17 2xRcRE reporter cells were co-transfected with HERV-K Con Rec (100 ng) and increasing amounts (0–800 ng) of untagged variants, keeping total DNA at 2,000 ng with the empty vector. The data were collected, analyzed, and normalized as described in Fig. 4C. The data represent the mean value ± SD from three independent experiments. Statistical significance was assessed by ordinary two-way ANOVA with Dunnett’s multiple comparisons *post hoc* test (*P < 0.05, **P < 0.01, and ****P < 0.0001). (**F**) Trans-dominant negative activity analysis using HA-tagged Rec variants under identical conditions as panel E. Cells were co-transfected with HERV-K Con Rec (100 ng) and increasing amounts (0–800 ng) of HA-tagged variants. The data were collected, analyzed, and normalized as described in Fig. 4C. Data represent the mean value ± SD from three independent experiments. Statistical significance was assessed by ordinary two-way ANOVA with Dunnett’s multiple comparisons *post hoc* test (*P < 0.05, **P < 0.01, ***P < 0.001, and ****P < 0.0001).

Since Rec 12q14.1 demonstrated a potent trans-dominant negative effect while having only two amino acid changes compared to the HERV-K Con Rec, we sought to determine if one or both changes were responsible for the trans-dominance. We thus generated two variant mutants of this Rec protein, as shown in Fig. 5A: Variant 1, which restored a glutamic acid at position 34 (del34E), and Variant 2, which changed the histidine at position 2 to the asparagine present in HERV-K Con Rec (H2N). The variant sequences were cloned into the MSCV vector upstream of the IRES-eBFP2 region, and HA-tagged versions of the original 12q14.1 and the two variants were also generated to enable analysis of protein expression. Western blot analysis of 293T/17 reporter cells using the HA-tagged versions of these constructs showed that all three proteins were well expressed (Fig. 5B).

The untagged constructs were then packaged into retroviral particles and used to transduce the RcRE-reporter cell line, in parallel with vectors that expressed the original 12q14.1 Rec and HERV-K Con Rec as a positive control. Rec activity was measured as described in Fig. 2B. This experiment revealed that Variant 1 showed functional activity similar to HERV-K Con Rec (Fig. 5C), whereas Variant 2 and the original 12q14.1 Rec were non-functional.

We also tested the activity of the HA-tagged 12q14.1 Rec variants. This was done by transfection of the HA-tagged plasmids into the RcRE-reporter cell line and measurement of GFP MFI by flow cytometry. Similar to the untagged constructs, HA-tagged Variant 1 showed Rec/RcRE functional activity, but the HA-tagged Variant 2 and the original HA-tagged 12q14.1 Rec did not (Fig. 5D). These results indicate that deletion of E34 alone abolishes Rec functional activity, whereas changing the asparagine to histidine at position 2 has minimal impact on function.

We next analyzed the trans-dominant negative activities of the two variants by co-expressing increasing amounts of each variant with a constant amount of HERV-K Con Rec as described above for Fig. 3. Variant 2 (E34del) exhibited a trans-dominant negative effect nearly identical to the original Rec 12q14.1 (Fig. 5E). The HA-tagged versions gave similar results (Fig. 5F). Notably, both tagged and untagged Variant 1 showed no trans-dominant negative effect, and expression of this variant increased the overall activity.

However, noticeably, the increase in activity with the non-tagged Rec Variant 1 protein was considerably less than with the HA-tagged protein construct (Fig. 5E and F). This is likely due to saturation of functional activity with higher plasmid doses. Figure S2, which is the same data plotted without normalization, shows that the baseline GFP MFIs are different. The baseline value with the non-tagged Rec appears to already be at a level of saturation that is reached by the HA-tagged Rec only at higher DNA concentrations.

Based on these experiments, we conclude that the glutamic acid at position 34 (E34) is essential for Rec functional activity, and that this deletion alone causes a trans-dominant negative phenotype.

### Functional analysis of Rec proteins from loci identified since the Subramanian 2011 study

**Fig 6:**
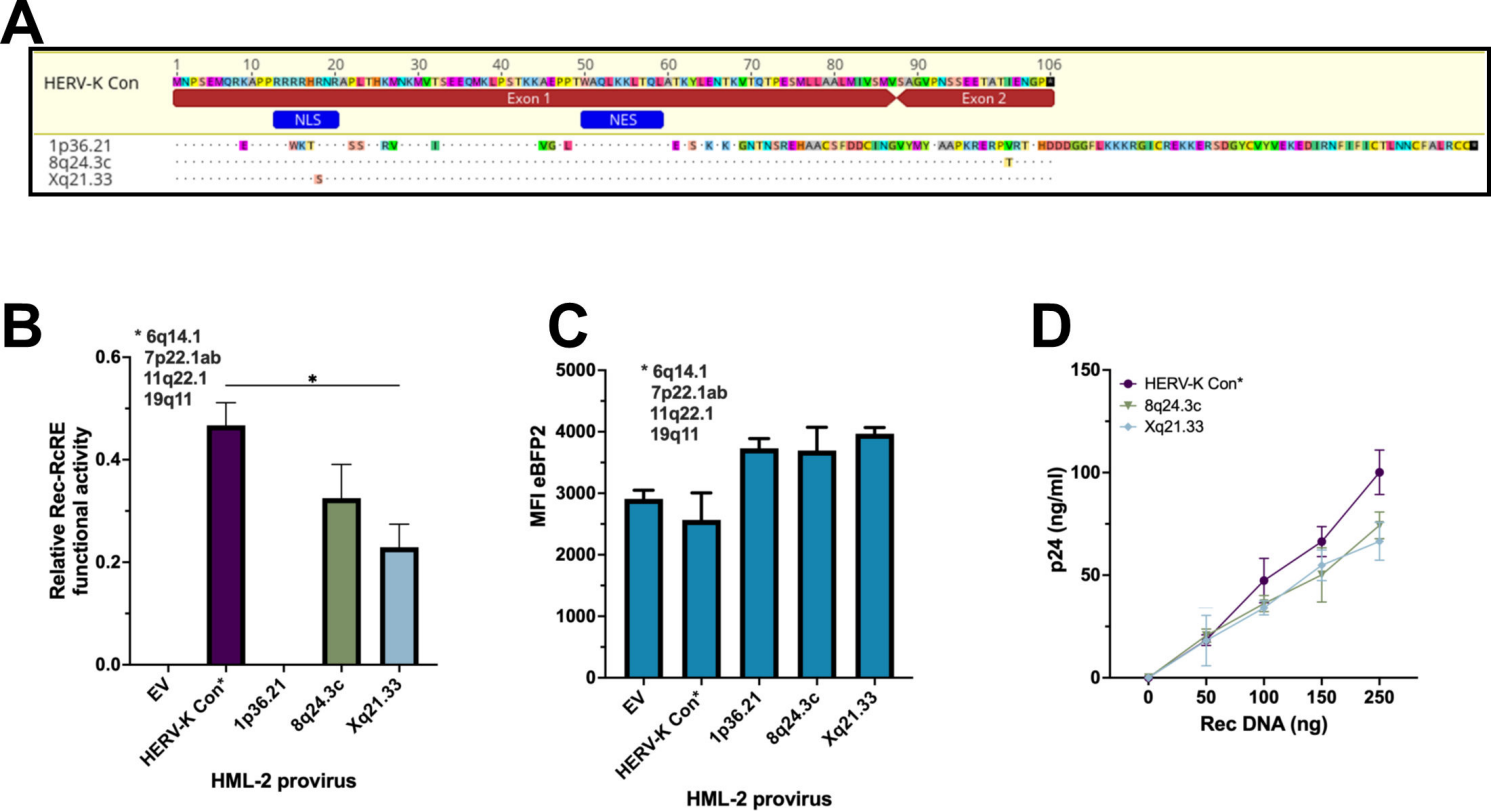
Functional analysis of HERV-K Rec proteins from proviruses identified since the Subramanian 2011 listing (5). (**A**) Sequence alignments of Rec proteins from newly identified proviruses relative to the HERV-K Con Rec sequence. The alignments include Rec sequences from 1p36.21, 8q24.3c, and Xq21.33 proviruses identified after the publication of reference 5. The HERV-K Con Rec sequence is shown as the reference at the top. Dots indicate amino acid identity with the HERV-K Con Rec consensus; differences are highlighted in color and shown as uppercase letters. Stop codons are indicated by black boxes with an asterisk (*). Position numbers correspond to the HERV-K Con Rec protein. (**B**) Quantification of Rec functional activity using the dual-color reporter assay by flow cytometry analysis performed 72 hours post-transduction. 293T/17 RcRE-reporter cells were transduced with vectors expressing the various Rec proteins and eBFP2. Rec activity was assessed by gating on mCherry-positive cells, selecting eBFP2-positive cells (indicating Rec vector expression), and measuring GFP and eBFP2 expression. Rec/RcRE functional activity was calculated as the ratio of GFP mean fluorescence intensity to eBFP2 mean fluorescence intensity. The HERV-K Con Rec sequence represents five identical Rec proteins (6q14.1, 7p22.1a, 7p22.1b, 11q22.1, and 19q11) (marked with an asterisk). The data are shown as the mean value ± SD from three independent experiments. Significant differences were assessed by ordinary one-way ANOVA with Tukey’s *post hoc* test (*P < 0.05). (**C**) eBFP2 expression levels from the transductions performed in panel **B**. The MFI of eBFP2 expressed from each of the individual transductions was determined using flow cytometry. Data are shown as mean ± SD from three independent experiments. (**D**) Validation of Rec activity using a p24 release assay. 293T/17 cells were co-transfected with equal amounts of a Gag-Pol-RcRE reporter vector (1,500 ng) and increasing amounts of functional Rec vectors (50, 100, 150, and 250 ng). An empty vector was used to normalize the total DNA input to 2,000 ng. Supernatants were collected at 72 hours post-transfection, and p24 was then measured by ELISA. The HERV-K Con Rec sequence represents five identical Rec 1013 proteins (6q14.1, 7p22.1a, 7p22.1b, 11q22.1, and 19q11) (marked with an asterisk). The data shown are the mean values ± SD from three independent experiments.

Three new type 2 proviral loci have been identified since the Subramanian compilation. Two (8q24.3c and Xq21.33) have been described as insertional polymorphic (42). The third (1p36.21) is absent from hg19 but is present in the hg38 genome assembly (43). The Rec proteins that can be expressed from these proviruses are shown in Fig. 6A, in an alignment to HERV-K Con Rec. The two polymorphic proviruses showed only a single amino acid change, whereas the third was highly mutated and C-terminally extended because of reading-frame shifts and other changes. We made retrovirus constructs for these three proteins and tested them in both transduction and transfection experiments, in comparison with HERV-K Con Rec. The results, shown in Fig. 6B and D, demonstrated that the highly mutated Rec protein (1p36.21) showed no functional activity, whereas the other two proteins functioned well, although not as well as the HERV-K Con Rec protein. Figure 6C shows eBFP2 expression levels in the transduced cells, confirming comparable transduction efficiencies. These findings further highlight that proteins with more than a few mutations relative to HERV-K Con Rec are unlikely to function at the post-transcriptional level. However, Rec encoded by new proviruses can easily be tested for function, using our reporters, to confirm or refute this.

## DISCUSSION

In this study, we show that 25 of the HERV-K type 2 proviruses in the human genome have open reading frames capable of producing full-length Rec proteins. However, only nine of these loci express proteins with post-transcriptional function. Among these, eight are insertionally polymorphic (6q14.1, 7p22.1a, 7p22.1b, 8p23.1a, 8q24.3c 11q22.1, 19p12b, and Xq21.33) in human populations. 19q11 is not polymorphic at the integration site, although it has been reported to be internally polymorphic (43–47). These functional loci all have nucleotide sequence changes in the *rec* gene relative to the prototypical HERV-K Con *rec* gene commonly used in published studies (43, 45–47). In five of these loci, the nucleotide changes are synonymous within the Rec ORF (6q14.1, 7p22.1a, 7p22.1b, 11q22.1, and 19q11), and they thus express a protein with the same amino acid sequence as HERV-K Con. For four of the loci (8p23.1a, 11q22.1, 8q24.3c, and Xq21.33), there are a few amino acid changes relative to HERV-K Con (see Fig. 1C; Fig. S3). None of the changes is in the known NLS and NES domains.

The evolutionary age of the HERV-K proviruses differs significantly. The relatively younger proviral insertions, such as those at loci 6q14.1 and 19p12b, are more likely to retain functional Rec proteins due to fewer accumulated mutations (1, 2). Conversely, older proviruses are typically heavily mutated, rendering their encoded Rec proteins non-functional, as seen for proviruses at several well-characterized loci (3, 5, 12). Importantly, one older provirus, the nearly intact locus 19q11, maintains the potential to produce functional Rec and other viral proteins despite its age, indicating some exceptions to this (7, 12, 18).

About half of the HERV-K proviruses are type 1 and contain a deletion in Env and the overlapping Rec region. This eliminates the ability to synthesize a Rec protein, making these proviruses unable to export their unspliced genome mRNA, which requires the help of an export protein, to express Gag and GagPol proteins. Additionally, the singly spliced mRNA, which encodes an N-terminally truncated Env protein, still retains an intron, so *env* expression would also be expected to be impaired (10). Thus, cells transcribing RNA from only type 1 viruses would be limited to expressing only the Np9 protein that is translated from a Rec-independent multiply spliced mRNA, unless the same cell expresses functional Rec from a type 2 HERV-K provirus. Similarly, type 2 proviruses lacking a functional Rec could also be complemented in this way to export all of their mRNAs with retained introns and express their viral structural proteins. This *trans*-complementation would be similar to our previous study, which showed that the HIV Rev protein can complement Rec function when supplied in *trans*, to enable nuclear export of endogenously expressed HERV-K RNAs with retained introns (28).

Several studies have implicated HERV-K in various malignancies and autoimmune disorders (13, 29, 30). For instance, a recent study reported robust transcription from certain HERV-K loci in lung cancer, highlighting the potential clinical significance (17, 48). Several published studies have also documented HERV-K expression in specific biological contexts. However, most of these studies have focused on the expression of Env proteins (49–51). Since the mRNA for the Env proteins retains an intron and would be expected to be dependent on Rec (10), functional Rec is likely expressed in the cells as well. Expression of Rec has also been observed in germ cells (26) and certain tumors such as melanomas (52). However, none of these studies examined whether functional Rec proteins were expressed. In addition to disease-associated expression, it has been demonstrated that Rec and Np9 transcripts are present in a range of normal human tissues, indicating that transcription from HERV-K loci is not restricted to pathological contexts (38). This finding underscores the importance of considering both physiological and disease-associated expression patterns when evaluating Rec function.

As mentioned in the Introduction, a previous study implicated HERV-K Rec proteins in cancer-related processes through interactions with the PLZF, leading to c-myc upregulation and enhanced cell proliferation (13). In addition, Rec protein interactions with the hSGT were reported to increase androgen receptor activity, potentially contributing to oncogenesis (30). It remains to be determined how many of the Rec proteins can interact with these proteins and cause these effects, since this may not require the protein to function in RNA expression. Activation of HERV-K and production of virus particles, which should include functional Rec expression, have also been implicated in melanoma progression (53). However, in one study, Rec was shown to form a regulatory feedback loop with the microphthalmia-associated transcription factor, potentially counteracting progression to a more invasive phenotype (54). This suggests that the effects of Rec in cancer may be context-dependent and not exclusively pro-oncogenic (for a review, see reference 55).

In addition to the few loci that can express a Rec protein that functions in post-transcriptional RNA regulation, we have determined that some Rec proteins show trans-dominant negative activity. The most striking example is the Rec encoded by provirus 12q14.1, which differs from the consensus by only two amino acid changes (N2H and E34del), yet potently inhibits functional Rec activity. This highlights that a few amino acid changes in retroviral regulatory proteins can significantly affect function, as shown previously for HIV Rev (56). Our mutational analyses revealed that reintroducing glutamic acid at position 34 restored export function. Interestingly, the deletion is not in a region known to be important for nuclear export, import, or dimerization, and thus it inhibits Rec function through an unknown mechanism (17, 57). This contrasts with the well-studied trans-dominant negative Rev protein (RevM10) that has mutations in the nuclear export signal (58–60).

The trans-dominant negative Rec proteins may serve as a natural “brake” in HERV-K replication and expression. This could potentially limit pathogenic HERV-K effects, and these Rec variants may thus have been positively selected during HERV-K evolution and spread. Additionally, the deletion of Rec in the type 1 HERV-K proviruses eliminates potential pathogenic effects resulting from Rec overexpression. However, since Rec can act in *trans*, if expressed from a type 2 virus that is active in the same cell, type 1 viruses do not need to encode Rec for replication. HERV-K proviruses have also been reported to produce viral particles capable of packaging and transmitting HERV-K-related sequences in cell culture (61). This highlights the potential for functional Rec proteins to contribute to the mobilization of endogenous retroviral elements under certain conditions.

Our discovery that active proviral copies of HERV-K can encode both active and inhibitory Rec proteins shows the complexity of Rec regulation. Levels of HERV-K expression and potential host cell effects of Rec activity will vary depending on which HERV-K loci are active, and it remains possible that Rec is an important factor in some human cancers. Further studies on the oncogenic properties of Rec and their relationship to the function of Rec as a post-transcriptional factor will be necessary to elucidate this.

## MATERIALS AND METHODS

### Identification of HERV-K HML-2 Rec ORFs

Human endogenous retrovirus K HML-2 proviral sequences (*n* = 91) were retrieved from the NCBI Nucleotide Database (GenBank) (see Table S4 for accession numbers) (5). Each HERV-K HML-2 genome was aligned to the annotated HERV-K Con reference genome using Geneious Prime software (version 2020.2, Biomatters Ltd., Auckland, New Zealand) to transfer annotated proviral features. Type 1 proviruses were identified based on the presence of the 292-bp deletion in the pol-env region and the GA-GT mutation in the 5′ splice site. Rec coding sequences from the identified type 2 proviruses lacking the 292-bp deletion were generated by extracting and joining the annotated Rec exons from each type 2 provirus. Sequence alignments were performed using the MUSCLE algorithm with default parameters as implemented in Geneious.

### Plasmids and vector construction

#### Reporter vector system

The dual-color Rec-RcRE reporter vector was adapted from the previously described Rev-RRE HIV reporter system (40, 41). The HIV RRE sequence was replaced with two tandem copies of the HERV-K RcRE (2xRcRE) while maintaining the same vector backbone and fluorescent protein reporters. The RcRE sequence was derived from a HERV-K LTR sequence previously deposited in GenBank (accession number AF179225.1) and synthesized by Integrated DNA Technologies (IDT, Coralville, IA, USA).

#### Retroviral vectors

For Rec protein expression, HERV-K Rec open reading frames were commercially synthesized (Genscript, Piscataway, NJ, USA) or generated as gBlocks (Integrated DNA Technologies). They were then cloned into murine stem cell virus-based retroviral vectors containing an IRES-eBFP2 cassette (pMSCV-IRES-eBFP2). Cloning was performed using standard restriction enzyme digestion (EcoRI, XhoI; New England Biolabs, Ipswich, MA, USA) followed by T4 DNA ligase-mediated ligation (Thermo Fisher Scientific, Waltham, MA, USA) or using the NEB Builder HiFi Assembly Cloning Kit (New England BioLabs). Each plasmid was assigned a unique identifier (pHRXXXX) as detailed in Table S1. Construct integrity was confirmed by Sanger sequencing (Eton Bioscience Inc).

To generate N-terminal HA-tagged Rec expression constructs, we PCR-amplified each Rec variant using a forward primer encoding a 5′ HA epitope tag (MYPYDVPDYA) and a Kozak consensus sequence immediately upstream of the HA tag start codon. This primer also contained a 17-bp overhang complementary to the MSCV vector backbone. The reverse primer annealed near the 3′ end of the Rec open reading frame and included a complementary overhang to the MSCV vector. PCR was performed using Phusion High-Fidelity DNA Polymerase (Thermo Fisher Scientific) with the following cycling conditions: initial denaturation, 94°C for 30 s; 25 cycles of 94°C for 15 s, 65°C for 30 s, and 68°C for 30 s; final extension, 68°C for 10 min. The resulting PCR products were cloned into a modified MSCV vector lacking the IRES-eBFP2 cassette using Gibson Assembly (NEB). All constructs were verified by Sanger sequencing. The primer sequences are listed in Table S2.

#### Mutational analysis constructs

Rec 12q14.1 variants were generated using synthetic double-stranded DNA fragments (gBlocks; Integrated DNA Technologies) and subsequently cloned into the pMSCV vector upstream of the IRES-eBFP2 cassette via Gibson assembly following the manufacturer’s protocol. Each plasmid was assigned a unique identifier (pHRXXXX) as detailed in Table S1. Construct integrity was confirmed by Sanger sequencing (Eton Bioscience Inc.).

### Cell culture and viral vector production

#### Cell maintenance

293T/17 cells were maintained in Iscove’s modified Dulbecco’s medium (IMDM; Gibco, Thermo Fisher Scientific) supplemented with 10% bovine calf serum (BCS, Life Technologies) and 50 µg/mL gentamicin (Gibco). Cells were cultured at 37°C in a humidified 5% CO_2_ incubator.

#### Retroviral vector production

Retroviral vectors expressing Rec variants were produced in 293T/17 cells. Briefly, 8 × 10^6^ cells were seeded in 15 cm plates containing 20 mL growth medium (IMDM, 10% BCS, and 50 µg/mL gentamicin). After 24 hours, cells were transfected using polyethyleneimine (PEI; Polysciences, Warrington, PA, USA, 1 mg/mL stock solution, pH 7.0) with a plasmid mixture containing 30 µg pMSCV-Rec construct, 12.84 µg MLV-Pol (pHIT) plasmid, and 5.16 µg VSV-G expression plasmid (pMD2.G, Addgene). The DNA was mixed with PEI at a ratio of 1:3 (DNA:PEI) in serum-free IMDM and incubated for 20 min at room temperature before adding to cells. Following a 6-hour incubation in serum-free IMDM without antibiotics, the transfection medium was replaced with fresh growth medium. Forty-eight hours post-transfection, the viral supernatant was collected and cleared by centrifugation (380 RCF, 4°C, 5 min) to remove cellular debris. The cleared supernatant was then aliquoted (1 mL per aliquot) and stored at −80°C until use. Lentiviral particles containing the tandem RcRE-reporter were generated similarly, except using psPAX2 (Addgene) as the packaging plasmid in a ratio of 12.84 µg psPAX2, 30 µg reporter plasmid, and 5.16 µg pMD2.G per 15 cm plate.

For viral titration, serial 10-fold dilutions were prepared in serum- and antibiotic-free culture medium. 293T/17 cells (3 × 10^4^) were seeded in 96-well plates 24 hours before transduction. Cells were transduced with 100 µL of diluted viral stocks containing 6 µg/mL DEAE-dextran (Sigma-Aldrich) and incubated for 6 hours at 37°C and 5% CO_2_. Following medium replacement, cells were cultured for 72 hours, then harvested and resuspended in PBS containing 5% BCS. mCherry or eBFP2 expression was quantified using an Attune NxT flow cytometer with autosampler (Thermo Fisher Scientific). Viral titers were calculated as transducing units per milliliter (TU/mL) based on the percentage of fluorescent protein-positive cells in the linear range of dilutions (between 1% and 20% positive cells).

### Generation of stable reporter cell line

A stable 293T/17 reporter cell line (293T/17: pNL4-3[GFP] [HERV-K-2xRcRE] [mCherry]) was established through lentiviral transduction. Briefly, 3 × 10^6^ 293T/17 cells were seeded in 10 cm dishes 24 hours before transduction. Cells were transduced at an MOI of 0.1 with the lentiviral reporter construct in serum- and antibiotic-free IMDM containing 6 µg/mL DEAE-dextran. Following a 6-hour incubation at 37°C and 5% CO_2_, the virus-containing medium was replaced with standard growth medium.

Transduced cells were subjected to fluorescence-activated cell sorting using a BD Influx System (BD Biosciences, San Jose, CA, USA). Single cells expressing the highest levels of mCherry were isolated and seeded into individual wells of 96-well plates containing IMDM supplemented with 20% BCS and 50 µg/mL gentamicin. After a 2-week expansion period, clonal populations were cryopreserved in growth medium containing 10% DMSO (Sigma-Aldrich). The established clonal cell line was subsequently evaluated for consistent and robust reporter gene expression. Functional assays were performed to confirm the responsiveness of the reporter system to the presence of functional Rec proteins, indicated by GFP expression upon Rec/RcRE interaction.

### Functional analysis of Rec variants

To assess Rec/RcRE functional activity, reporter 293T/17 cells (3 × 10^4^ cells/well) were seeded in 96-well plates 24 hours before transduction. Cells were transduced at an MOI of 1 with retroviral vectors expressing individual Rec variants in serum- and antibiotic-free RPMI containing 6 µg/mL DEAE-dextran. Following a 6-hour incubation at 37°C and 5% CO_2_, virus-containing medium was replaced with standard growth medium. At 72 hours post-transduction, cells were harvested by trypsinization using 0.25% Trypsin-EDTA (Gibco), resuspended in PBS containing 5% BCS, and analyzed by flow cytometry. Single-color controls were included for proper compensation while measuring mCherry, GFP, and eBFP2 expression.

### Western blot analysis

To analyze protein expression levels, reporter 293T/17 cells were transfected with 2 µg of MSCV plasmids expressing either functional or non-functional Rec variants using Lipofectamine 3000 (Invitrogen, Thermo Fisher Scientific) according to the manufacturer’s protocol. At 72 hours post-transfection, cells were harvested and split for parallel flow cytometry analysis and Western blot.

For Western blot samples, cells were washed three times with cold PBS and lysed in buffer containing 50 mM Tris, pH 7.4, 150 mM NaCl, 1.5% SDS, and protease inhibitors. Lysates were passed through a 21G needle (BD), heat-treated (90°C, 10 min), and cleared by centrifugation (14,000 RCF, 10 min) using a microcentrifuge (Eppendorf, Hamburg, Germany). Samples were prepared for SDS-PAGE by combining 50 µL lysate with NuPAGE LDS Sample buffer (4×) (Invitrogen) and NuPAGE Sample Reducing Agent (10×) (Invitrogen), followed by heating at 70°C for 10 min.

Proteins were separated on Novex 4%–12% Bis-Tris gels (Invitrogen) at 120 V for 1.5 hours using an electrophoresis system and transferred to PVDF membranes at 30 V for 1 hour at 4°C. Membranes were blocked with 5% BSA (Sigma-Aldrich) in TBS (1 hour, room temperature) and incubated overnight at 4°C with primary antibodies (Mouse anti-HA Tag, Cell Signaling Technology; 1:30,000 dilution and Rabbit anti-Beta Tubulin, Cell Signaling Technology; 1:2,000 dilution) diluted in TBS-T (TBS + 0.1% Tween 20) containing 5% BSA.

Membranes were washed three times with TBS-T (10 min each) and were incubated with IRDye-conjugated secondary antibodies (donkey anti-mouse IRDye 800CW, LI-COR Biosciences; 1:30,000 dilution and goat anti-rabbit IRDye 680RD; LI-COR Biosciences, 1:5,000) for 1 hour at room temperature. After three additional washes with TBS-T, protein detection was performed using a LICOR Odyssey CLx scanner, and band intensities were quantified using Image Studio Software (version 5.2, LI-COR Biosciences). For quantification, Rec protein band intensities were normalized to β-tubulin loading controls from the same lanes. Antibodies used are listed in Table S3.

To normalize functional activity to protein expression, we divided the average mean fluorescence intensity, measured by flow cytometry, by the relative protein abundance of each Rec variant as detected by Western blotting of the HA-tagged protein. To accurately compare protein levels between samples, HA-tag signals were first normalized to β-tubulin as a loading control. The resulting normalized activity was calculated as follows:


$$Normalized\;MFI=\frac{Mean\;Fluorescence\;Intensity\;\left(MFI\right)}{\left(\frac{HA\;Expression}{\beta \;-\;Tubulin\;Expression}\;\right)},$$

where MFI is the average mean fluorescence intensity measured by flow cytometry, representing functional activity; HA is the average HA-tagged Rec protein level measured by Western blot; and β-tubulin is the loading control used to normalize the HA signal across samples.

### Flow cytometry

Cells were harvested by trypsinization and resuspended in PBS containing 5% BCS. Samples were analyzed using an Attune NxT flow cytometer with autosampler (Thermo Fisher Scientific). For each sample, a minimum of 30,000 events were collected. Single-color controls were used for compensation using the automated compensation feature of the FlowJo software. Data analysis was performed using FlowJo version 10.6.1 (FlowJo, LLC, BD Biosciences).

The analysis workflow included initial gating on single cells using forward and side scatter properties (FSC-A vs SSC-A) followed by doublet exclusion (FSC-A vs FSC-H). Gates for mCherry, eBFP2, and GFP-positive populations were established using untransduced 293T/17 cells as negative controls. All subsequent analyses were performed on the mCherry-positive population.

Relative Rec/RcRE functional activity was quantified as follows. For Fig. 2B and 5C, it was calculated as the ratio of GFP to eBFP2 mean fluorescence intensity. For all other figures, it was measured as GFP MFI from mCherry-positive populations.

### Trans-dominant negative Rec assays

To measure the effect of co-expressing the functional HERV-K Con with the selected non-functional Rec proteins, 2.5 × 10^5^ reporter 293T/17 cells were seeded in 24-well plates and transfected with 100 ng of the pMSCV-HERV-K Con Rec, and either 0, 200, 400, or 800 ng of pMSCV-IRES-eBFP2 vector expressing each of the four test non-functional Recs. Transfections were performed using Lipofectamine 3000 Transfection Reagent (Invitrogen) according to the manufacturer’s protocol. For each well, DNA was mixed with 1 µL of the P3000 and 1.5 µL of Lipofectamine 3000 in 50 µL of Opti-MEM. Variable amounts of an empty pMSCV plasmid were added to maintain a constant DNA mass of 2 µg in each transfection. Cells were incubated for 72 hours and subjected to flow cytometry to measure the expression of mCherry and GFP. The same method was used to test the effects of co-expressing HERV-K Con Rec with the Rec 12q14.1 point mutants.

### p24 assays

To assess Rec-mediated viral protein expression, 2.3 × 10^5^ 293T/17 cells were seeded per well in 12-well plates. Cells were transfected with a constant amount (1,500 ng) of the Gag-Pol-RcRE reporter vector and increasing amounts of Rec expression plasmids (50, 100, 150, and 250 ng). Transfections were performed using PEI as described above. At 72 hours post-transfection, culture supernatants were harvested, centrifuged at 300 × *g* for 5 min to remove cellular debris, and analyzed for p24 capsid protein using an in-house enzyme-linked immunosorbent assay, as previously described (62).

Briefly, 96-well plates (Nunc MaxiSorp, Thermo Fisher Scientific) were coated with 100 µL of anti-p24 monoclonal antibody (NIH AIDS Reagent Program) at a 1:10,000 dilution in PBS overnight at 37°C. Plates were washed five times with PBS and blocked with 200 µL of PBS containing 5% blocking buffer (PBS with 5% BCS) for 1 hour at 37°C. After washing, 100 µL of culture supernatants or p24 standards (NIH AIDS Reagent Program) ranging from 12.5 to 1,600 pg/mL were added to the wells and incubated for 2 hours at 37°C. Plates were washed and incubated with 100 µL of mouse anti-p24 polyclonal antibody (NIH AIDS Reagent Program) at a 1:10,000 dilution for 1 hour at 37°C. After washing, 100 µL of horseradish peroxidase-conjugated goat anti-mouse IgG (Abcam) at a 1:20,000 dilution was added and incubated for 30 min at 37°C. Plates were washed and developed with 100 µL of substrate for 30 min at room temperature in the dark. The reaction was stopped with 50 µL of 1 N H_2_SO_4_, and absorbance was measured at 450 nm using a microplate reader (BioTek Synergy HTX, BioTek Instruments). A four-parameter logistic regression standard curve was generated, and p24 concentrations in the samples were interpolated from this standard curve. Each sample was assayed in duplicate, and the average value was reported.

### Statistical analysis

Statistical analyses were performed using GraphPad Prism software (version 9.0; GraphPad Software, San Diego, CA, USA). Data are presented as mean ± standard deviation, derived from at least three independent experiments unless otherwise indicated in the figure legends. For comparisons involving more than two experimental groups, statistical significance was determined using ordinary one-way ANOVA followed by Tukey’s multiple comparisons test or by non-parametric one-way ANOVA with Dunn’s multiple comparisons *post hoc* test, depending on the outcome of data normality assessments. For comparisons between two groups, either an unpaired two-tailed Student’s *t*-test or the non-parametric Mann–Whitney *U* test was used, as appropriate. Trans-dominant negative activity assays, involving multiple conditions across different expression levels, were analyzed using ordinary two-way ANOVA followed by Dunnett’s multiple comparisons test against a single control (functional Rec alone), assuming pooled variance. For experiments involving multiple comparisons, *P*-values were adjusted using the Benjamini-Hochberg procedure to control the false discovery rate. A *P*-value of less than 0.05 was considered statistically significant. Significance thresholds used throughout the manuscript were defined as follows: *P < 0.05, **P < 0.01, ***P < 0.001, and ****P < 0.0001. The specific statistical tests applied to individual experiments are indicated in the corresponding figure legends. Graphs were prepared using GraphPad Prism.

## Data Availability

The sequences of HERV-K proviral loci analyzed in this study were previously deposited in NCBI GenBank (https://www.ncbi.nlm.nih.gov/nucleotide/) and are readily available by clicking on the hyperlinks provided in Table S4. The predicted Rec cDNA and protein sequences from these loci are provided as FASTA files in File S1 and S2, respectively. All data generated or analyzed during this study are included in this published article and its supplemental material.

## SUPPLEMENTAL MATERIAL

File S1 jvi.01515-25-s0001.txt FASTA files of Rec cDNA sequences.

File S2 jvi.01515-25-s0002.txt FASTA files of Rec protein sequences.

Supplemental figures jvi.01515-25-s0003.pdf Figures S1 to S3.

Supplemental tables jvi.01515-25-s0004.pdf Tables S1 to S5.

ASM does not own the copyrights to Supplemental Material that may be linked to, or accessed through, an article. The authors have granted ASM a non-exclusive, world-wide license to publish the Supplemental Material files. Please contact the corresponding author directly for reuse.
