# Supplementary material for: Characterization of HERV-K (HML-2) Rec proteins encoded in the human genome and their post-transcriptional function
Source: J Virol. 2025 Nov 18;99(12):e01515-25. doi: 10.1128/jvi.01515-25 (PMC12724133; doi:10.1128/jvi.01515-25)
Supplement: Supplemental figures — Figures S1 to S3. [file jvi.01515-25-s0003.pdf]

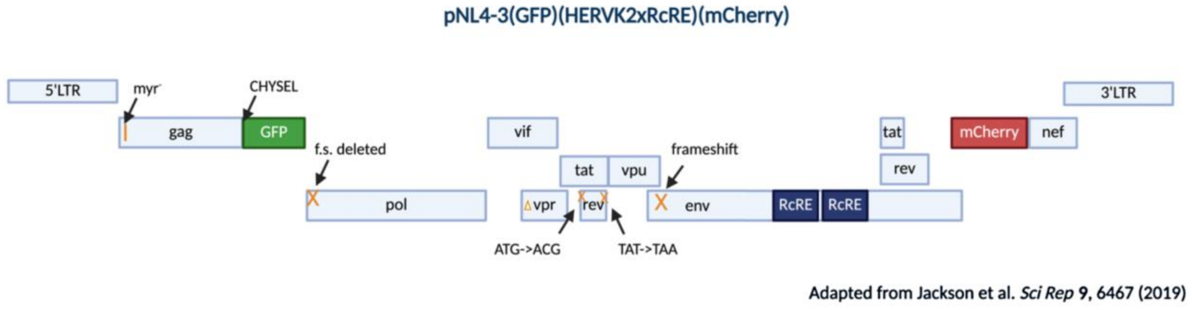

Figure S1: The dual-color Rec-RcRE reporter vector

The dual-color Rec-RcRE reporter vector was adapted from the previously described Rev-RRE HIV reporter system (*Jackson et al. Sci. Rep 9 6467 (2019)*). In this modified construct, the HIV RRE was replaced with two tandem copies of the HERV-K RcRE, while retaining the same lentiviral backbone and fluorescent reporter configuration. The RcRE sequence was derived from GenBank accession AF179225.1. The vector is based on the HIV-1 NL4-3 genome, with the following modifications: mutation of the gag myristoylation signal, truncation of gag with insertion of a CHYSEL sequence and eGFP, deletion of the frameshift site, deletion in the vpr gene, inactivation of the rev start codon and insertion of a stop codon in exon 1, frameshift mutation in env, flanking of the RcRE region with XmaI and XbaI restriction sites, and replacement of nef with a second fluorescent protein (mCherry). This system enables functional testing of Rec-RcRE–mediated mRNA export and expression.

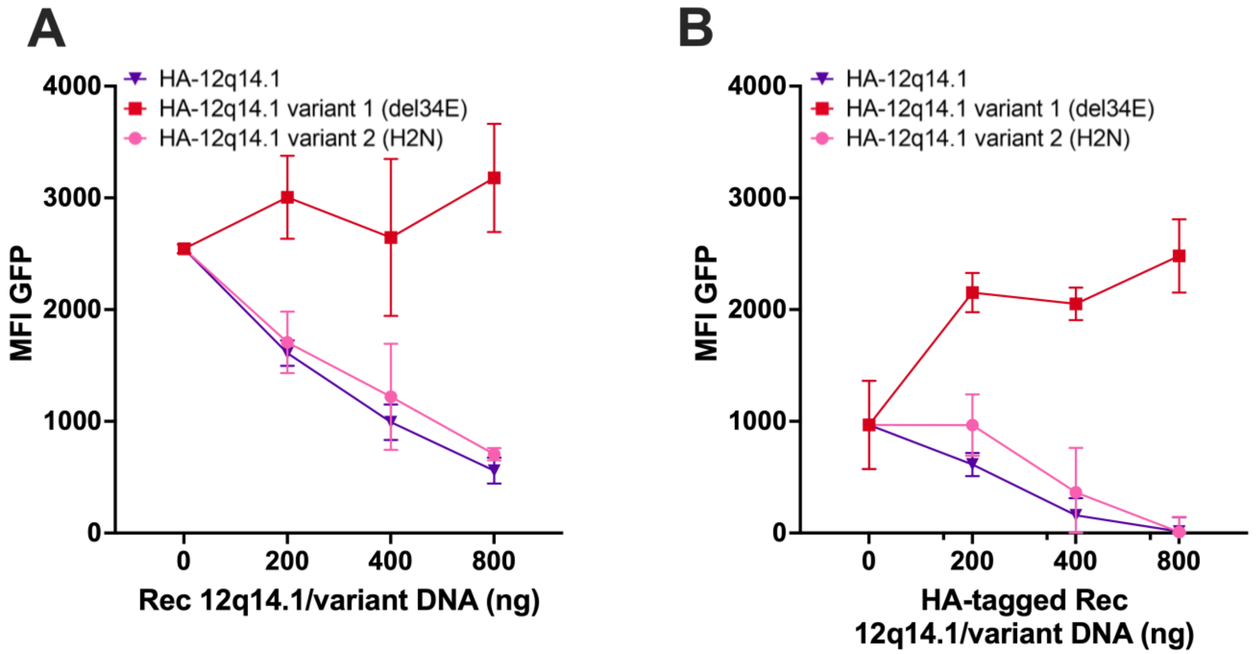

Figure S2. Trans-dominant negative activity analysis using untagged and HA tagged Rec variants.

This figure plots the non-normalized data that is plotted as normalized data in Figure 5E and 5F.(A) 293T/17 2xRcRE reporter cells were co-transfected with HERV-K Con Rec (100 ng) and increasing amounts (0–800 ng) of untagged variants. (B) 293T/17 2xRcRE reporter cells were co-transfected with HA-HERV-K Con Rec (100 ng) and increasing amounts (0–800 ng) of HA-tagged variants. In all cases total DNA was kept at 2000 ng with the addition of empty vector. Mean fluorescence intensity (MFI) of GFP expression above background was quantified by flow cytometry at 72 hours post-transfection. Data are representative of three independent experiments; error bars represent SD

Non-tagged Rec exhibited higher baseline activity and reached a plateau beyond 200 ng of added variant 1, consistent with saturation of its activity. In contrast, HA-tagged Rec showed lower baseline expression and an initial dose response with added HA-variant 1, which later reached saturation at about the same level of GFP expression as the untagged variant 1.

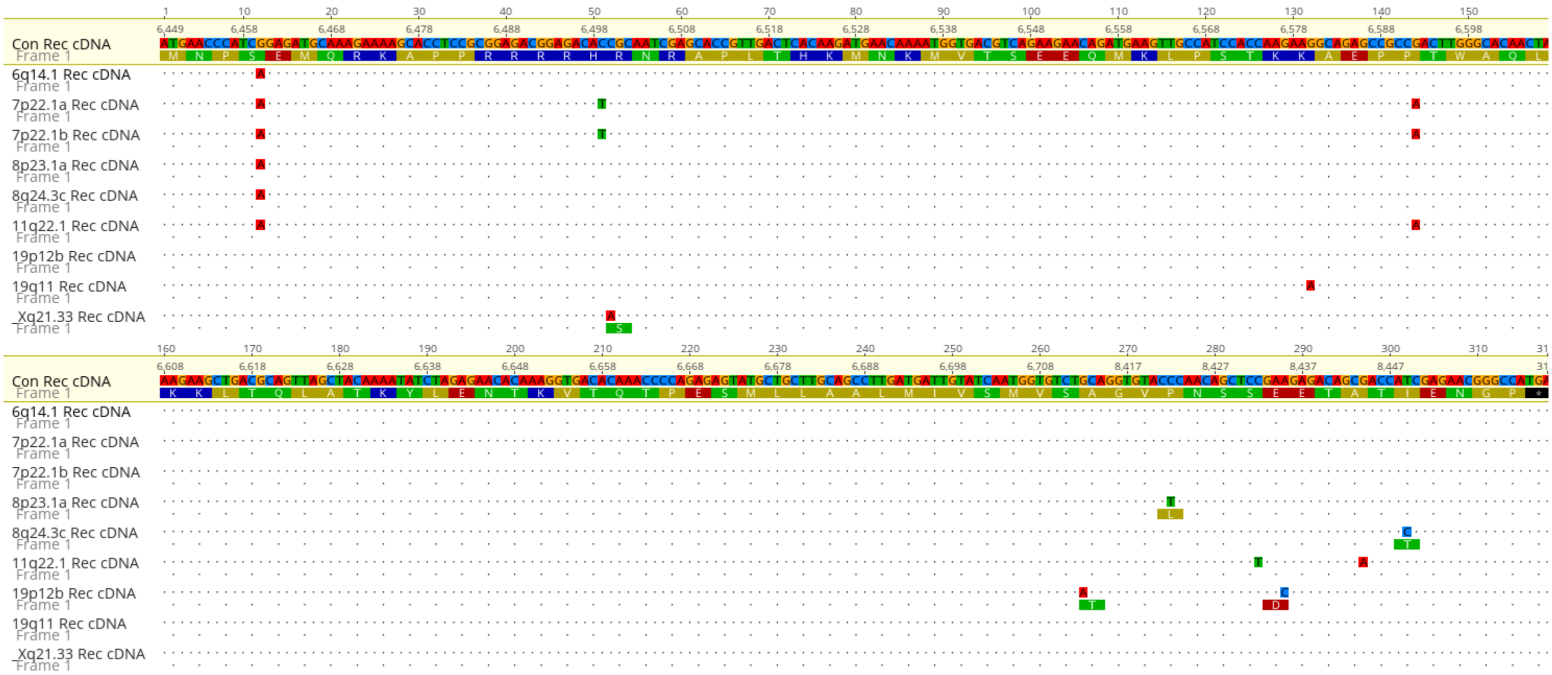

Figure S3. Alignment of *rec* cDNA sequences encoding Rec proteins that function in the dual-color assay.

All sequences are aligned to the nucleotide sequence of the cDNA encoding HERV-K Con Rec. For each sequence the top line represents the nucleotide sequence and the bottom line the translated protein. Dots indicate identity to the HERV-K sequence. The square boxes show nucleotide changes. The rectangular boxes show amino acid changes. The top row of numbers refers to the nucleotide positions the cDNA. The second row of numbers refers to nucleotide positions in the HERV-K Con provirus.
