# Supplementary material for: Characterization of HERV-K (HML-2) Rec proteins encoded in the human genome and their post-transcriptional function
Source: J Virol. 2025 Nov 18;99(12):e01515-25. doi: 10.1128/jvi.01515-25 (PMC12724133; doi:10.1128/jvi.01515-25)
Supplement: Supplemental tables — Tables S1 to S5. [file jvi.01515-25-s0004.pdf]

Table S1. List of the plasmids used in this study.

Packaging plasmids and plasmid backbone vectors

Construct	Plasmid number
pHIT (MLV packaging plasmid)	pHR1854
psPAX2 (HIV packaging plasmid)	pHR5691
pMD2.G (VSV-G)	pHR5693
NL4-3(GFP)(HERV-K-2xRcRE)(mCherry)	pHR5839
pMSCV-IRES-eBFP2	pHR5302
pMSCV-IRES	pHR6896

Rec-expression plasmids

HML-2 Rec	Plasmid number
1q24.1	pHR6338
1p36.21	pHR7196
3p12.3	pHR6339
3q21.2	pHR6340
4p16.1a	pHR6341
4p16.1b	pHR6342
4p16.3b	pHR6343
4q35.2	pHR6344
5p13.3	pHR6345
5q33.1	pHR6346
6q14.1	pHR6347
7p22.1ab	pHR6465
8p23.1a	pHR6348
8p23.1b	pHR6349

HML-2 Rec	Plasmid number
8p23.1c	pHR6350
8p23.1d	pHR6351
8q24.3c	pHR7194
10p14	pHR6352
11p15.4	pHR6354
12p11	pHR6359
12q14.1	pHR6355
12q14.1 variant 1	pHR6463
12q14.1 variant 2	pHR6464
19p12b	pHR6856
19q13.42	pHR6360
Xq21.33	pHR7192
Xq28b	pHR6357

HA-tagged Rec expression plasmids

HML-2 Rec	Plasmid number
HA-3q21.2	pHR6912
HA-5p13.3	pHR6908
HA-6q14.1	pHR6954
HA-7p22.1ab	pHR6942
HA-8p23.1a	pHR6944
HA-10p14	pHR6910
HA-12q14.1	pHR6952
HA-12q14.1 variant 1	pHR6950
HA-12q14.1 variant 2	pHR6956
HA-19p12b	pHR6948

Table S2. Primers used to generate N-terminally HA-tagged Rec cloned into a modified pMSCV plasmid using Gibson Assembly.

Primer	Sequence
5' primer	TCACTCCTTCTCTAGGCGCCGGAATTCACCATGTACCCATACGATGT TCCAGATTACGCT
3' primer	CATCGAGAACGGGCCATGACTCGAGCGGGATCAATTCCG

Table S3. List of antibodies used for Western blotting

Name	Manufacturer	Catalog number	Dilution
Mouse HA Tag Monoclonal Antibody (2-2.2.14)	Invitrogen	26183	1:30 000
Beta Tubulin Rabbit Polyclonal Ab	Proteintech	10094-1-AP	1:2000
Donkey anti-mouse IRDye 800CW	Licor	926-32212	1:30 000
Goat anti-rabbit IRDye 680RD	Licor	925-68071	1:5000

Table S4: HERV-K loci analyzed in this study

(adapted from Kyriakou and Magiorkinis, 2025)

	Chromosome locus	Alias	Genomic Coordinates (hg38)	Genbank accession number	Reference
1	1p34.3	-	chr1:36488984-36491127	JN675008	Subramanian et al. 2011
2	1p36.21#	1p36.21(327)	chr1:13012447-13021988	no number	Bendall et al. 2019
3	1p36.21b	HERV-K(OLDAL023753), K6, K76, 1p36.21(362)	chr1:13206972-13216513	JN675010	Reus et al. 2001
4	1p36.21c	K6,K76, 1p36.21(326)	chr1:13352736-13362257	JN675011	Hughes & Coffin 2001
5	1q21.3	-	chr1:150632808-150635885	JN675012	Subramanian et al. 2011
6	1q32.2	-	chr1:207635112-207639291	JN675016	Subramanian et al. 2011
7	1q24.1	K12	chr1:166605366-166611021	JN675015	Romano et al. 2006
8	3p12.3	-	chr3:75551314-75559999	JN675019	Subramanian et al. 2011
9	3q21.2	-	chr3:125890459-125899596	JN675023	Sugimoto et al. 2001
10	4p16.1a	K17b, 4p16.1(916)	chr4:9121786-9131367	JN675026	Romano et al. 2006
11	4p16.1b	K50c, 4p16.1(976)	chr4:9657956-9667550	JN675027	Macfarlane & Simmonds 2004
12	4p16.3b	K77, 4p16.3(374)	chr4:3977324-3986912	JN675029	Romano et al. 2006

13	4q13.2	-	chr4:68597991-68603505	JN675030	Subramanian et al. 2011
14	4q35.2	-	chr4:190106259-190113546	JN675033	Subramanian et al. 2011
15	5p13.3[P]	HERV-K104, K50d	chr5:30486653-30496098	AF164612, JN675035	Barbulescu et al. 1999
16	5q33.2	K18b	chr5:154635953-154644655	JN675036	Romano et al. 2006
17	6p11.2	K23	chr6:60654987-60660975	JN675038	Romano et al. 2006
18	6p21.1	K(OLDAL035587), KOLD35587	chr6:42893671-42903629	JN675039	Reus et al. 2001
19	6p22.1	K(OLDAL121932), K69, K20	chr6:28682591-28692958	JN675040	Reus et al. 2001
20	6q14.1[P]	HERV-K109, K(C6), ERVK-9	chr6:77716945-77726366	AF164615, JN675041	Barbulescu et al. 1999
21	7p22.1a[P]	HERV-K108L, K(HLM-2.HOM), K(C7), ERVK-6	chr7:4582426-4591897	JN675043	Mayer et al. 1999, Barbulescu et al. 1999
22	7p22.1b[P]	HERV-K108R, K(HLM-2.HOM), K(C7), ERVK-6	chr7: 4590930-4600400	JN675044	Mayer et al. 1999, Barbulescu et al. 1999
23	8p22	-	chr8:17907693-17916431	JN675048	Subramanian et al. 2011
24	8p23.1a[P]	HERV-K115, ERVK-8, 8p23.1(775)	chr8:7497875-7507337	AY037929, JN675049	Turner et al. 2001
25	8p23.1b	K27, 8p23.1(878)	chr8:8197178-8206699	JN675050	Hughes & Coffin 2001
26	8p23.1c	8p23.1(261)	chr8:12216461-12225988	JN675051	Hughes & Coffin 2001
27	8p23.1d	KOLD130352, 8p23.1(283)	chr8:12458983-12468498	DQ112134, JN675052	Hughes & Coffin 2001

28	8q11.1	K70, K43	chr8:46264028-46272039	JN675053	Romano et al. 2006
29	8q24.3b	8q24.3(514)	chr8:145021244-145028834	JN675055	Subramanian et al. 2011
30	8q24.3c#P	8q24.3(404)	chr8:144860784-144860789 (preintegraton site)	KU054255	Wildschutte et al. 2016
31	9q34.3	K30	chr9:136780314-136789776	JN675056	Hughes & Coffin 2001
32	9q34.11	K31, DE7, ERVK16	chr9:128850236-128857457	JN675057	Hughes & Coffin 2001
33	10p14	K(C11a), K33, ERVK-16	chr10:6824179-6833641	JN675059	Costas et al. 2001
34	10q24.2	HERV-K128, ERVK-17, c10_B	chr10:99820812-99827988	JN675060	Macfarlane & Simmonds 2004
35	11p15.4	K7, ERVK3-4, 11p15.4(376)	chr11:3447426-3456979	DQ112131, JN675061	Romano et al. 2006
36	11q12.1	-	chr11:58999975-59005723	JN675062	Subramanian et al. 2011
37	11q12.3	K(OLDAC004127), 11q12.3(255)	chr11:62375545-62383091	JN675063	Reus et al. 2001
38	11q22.1P	HERV-K118, K(C11c), K36, ERVK-25	chr11:101695063-101704528	JN675064	Costas et al. 2001
39	12p11.1	K50e	chr12:34619620-34629282	JN675066	Romano et al. 2006
40	12q14.1P	HERV-K119, K(C12), K41, ERVK-21	chr12:58327459-58336915	JN675068	Costas et al. 2001
41	12q24.33	K42	chr12:133090536-133096478	JN675070	Romano et al. 2006
42	15q25.2	-	chr15: 84160268-84163612	JN675073	Subramanian et al. 2011
43	17p13.1	-	chr17:8056337-8063901	JN675075	Subramanian et al. 2011

44	19p12a	K52, 19p12(061)	chr19:20276591-20286703	JN675076	Hughes & Coffin 2001
45	19p12b[P]	HERV-K113, De1, ERVK26, 19p12(184)	chr19: 21658734-21658740<br>chr19_GL383575.2_alt: 10454-19925	AY037928, JN675077	Turner et al. 2001
46	19q11	HERV-K132, K(C19), ERVK-19	chr19:27637590-27646453	JN675080	Tonjes et al. 1999
47	19q13.12a	19q13.12(525)	chr19:35572305-35576532	JN675081	Subramanian et al. 2011
48	19q13.12b	K(OLDAC012309), KOLD12309, K50F, 19q13.12(767)	chr19:37106647-37116164	DQ112151, JN675082	Reus et al. 2001
49	19q13.41	-	chr19: 52745023-52750455	JN675083	Hughes & Coffin 2001
50	19q13.42	LTR13	chr19: 53359095-53364791	JN675084	Subramanian et al. 2011
51	20q11.22	K(OLDAL136419), K59	chr20: 34126944-34136578	DQ112105, JN675085	Hughes & Coffin 2001
52	22q11.23	K(OLDAP000345), KOLD345, ERVK-32	chr22: 23536062-23548428	JN675088	Hughes & Coffin 2001
53	Xq11.1	-	chrX: 62740079-62742584	JN675090	Subramanian et al. 2011
54	Xq21.33[#P]	De9	chrX: 94351605-94351609 (preintegraton site)	KU054272	Wildschutte et al. 2016
55	Xq28b	K63, Xq28b(483)	chrX: 154608423-154615762	JN675093	Macfarlane & Simmonds 2004
56	Yp11.2	-	chrY: 6958400-6965343	JN675094	Subramanian et al. 2011
57	Yq11.23a	Yq11.23(410)	chrY: 24251690-24254888	JN675095	Subramanian et al. 2011
58	Yq11.23b	Yq11.23(555)	chrY: 25415255-25418454	JN675096	Subramanian et al. 2011

Legend to Table S4

# indicates proviruses identified after publication of the comprehensive HML-2 catalog by Subramanian et al. (2011)

P indicates insertionally polymorphic proviruses

Red color indicates that the Rec sequence from this locus was tested in this study

Blue color indicates hyperlink to sequences

References for Table S4

Barbulescu, M., G. Turner, M. I. Seaman, A. S. Deinard, K. K. Kidd, and J. Lenz. 1999. 'Many human endogenous retrovirus K (HERV-K) proviruses are unique to humans', *Curr Biol*, 9: 861-8.

Bendall, M. L., M. de Mulder, L. P. Iñiguez, A. Lecanda-Sánchez, M. Pérez-Losada, M. A. Ostrowski, R. B. Jones, L. C. F. Mulder, G. Reyes-Terán, K. A. Crandall, C. E. Ormsby, and D. F. Nixon. 2019. 'Telescope: Characterization of the retrotranscriptome by accurate estimation of transposable element expression', *PLoS Comput Biol*, 15: e1006453.

Costas, J. 2001. 'Evolutionary dynamics of the human endogenous retrovirus family HERV-K inferred from full-length proviral genomes', *J Mol Evol*, 53: 237-43.

Hughes, J. F., and J. M. Coffin. 2001. 'Evidence for genomic rearrangements mediated by human endogenous retroviruses during primate evolution', *Nat Genet*, 29: 487-9.

Kyriakou, E., and G. Magiorkinis. 2025. 'Compilation of all known HERV-K HML-2 proviral integrations', *Mob DNA*, 16: 21.

Macfarlane, C., and P. Simmonds. 2004. 'Allelic variation of HERV-K(HML-2) endogenous retroviral elements in human populations', *J Mol Evol*, 59: 642-56.

Mayer, J., M. Sauter, A. Rácz, D. Scherer, N. Mueller-Lantzsch, and E. Meese. 1999. 'An almost-intact human endogenous retrovirus K on human chromosome 7', *Nat Genet*, 21: 257-8.

Reus, K., J. Mayer, M. Sauter, H. Zischler, N. Müller-Lantzsch, and E. Meese. 2001. 'HERV-K(OLD): ancestor sequences of the human endogenous retrovirus family HERV-K(HML-2)', *J Virol*, 75: 8917-26.

Romano, C. M., R. F. Ramalho, and P. M. Zanotto. 2006. 'Tempo and mode of ERV-K evolution in human and chimpanzee genomes', *Arch Virol*, 151: 2215-28.

Subramanian, R. P., J. H. Wildschutte, C. Russo, and J. M. Coffin. 2011. 'Identification, characterization, and comparative genomic distribution of the HERV-K (HML-2) group of human endogenous retroviruses', *Retrovirology*, 8: 90.

Sugimoto, J., N. Matsuura, Y. Kinjo, N. Takasu, T. Oda, and Y. Jinno. 2001. 'Transcriptionally active HERV-K genes: identification, isolation, and chromosomal mapping', *Genomics*, 72: 137-44.

Tönjes, R. R., F. Czauderna, and R. Kurth. 1999. 'Genome-wide screening, cloning, chromosomal assignment, and expression of full-length human endogenous retrovirus type K', *J Virol*, 73: 9187-95.

Turner, G., M. Barbulescu, M. Su, M. I. Jensen-Seaman, K. K. Kidd, and J. Lenz. 2001. 'Insertional polymorphisms of full-length endogenous retroviruses in humans', *Curr Biol*, 11: 1531-5.

Wildschutte, J. H., Z. H. Williams, M. Montesion, R. P. Subramanian, J. M. Kidd, and J. M. Coffin. 2016. 'Discovery of unfixed endogenous retrovirus insertions in diverse human populations', *Proc Natl Acad Sci U S A*, 113: E2326-34.

Table S5. Characteristics of the tested Rec proteins

Provirus	Rec ORF length (nt)	Rec ORF length (aa)	Functional in Assay	Trans-dominant negative activity
1p36.21	471	157	No	not tested
1q24.1	318	106	No	not tested
3p12.3	318	106	No	not tested
3q21.2	318	106	No	No
4p16.1a	318	106	No	not tested
4p16.1b	468	156	No	not tested
4p16.3b	468	156	No	not tested
4q35.2	306	102	No	not tested
5p13.3	318	106	No	Yes
5q33.2	318	106	No	not tested
6q14.1	318	106	Yes	N/A
7p22.1a	318	106	Yes	N/A
7p22.1b	318	106	Yes	N/A
8p23.1a	318	106	Yes	N/A
8p23.1b	318	106	No	not tested
8p23.1c	318	106	No	not tested
8p23.1d	318	106	No	not tested
8q24.3c	318	106	Yes	not tested
10p14	318	106	No	Yes
11p15.4	318	106	No	not tested
11q22.1	318	106	Yes	N/A
12p11.1	321	107	No	not tested
12q14.1	315	105	No	Yes
19p12b	318	106	Yes	N/A
19q11	318	106	Yes	N/A
19q13.42	315	105	No	not tested
Xq21.33	318	106	Yes	not tested
Xq28b	318	106	No	not tested

Legend to Table S5: N/A- not applicable
